# The burden of cancers associated with HIV in the South African public health sector, 2004–2014: a record linkage study

**DOI:** 10.1186/s13027-019-0228-7

**Published:** 2019-05-03

**Authors:** Tafadzwa Dhokotera, Julia Bohlius, Adrian Spoerri, Matthias Egger, Jabulani Ncayiyana, Victor Olago, Elvira Singh, Mazvita Sengayi

**Affiliations:** 1 National Cancer Registry, National Health Laboratory Service, Johannesburg, South Africa; 2 Division of Epidemiology and Biostatistics, School of Public Health, University of the Witwatersrand, Johannesburg, South Africa; 3 Institute of Social and Preventive Medicine (ISPM), University of Bern, Bern, Switzerland; 4 Centre for Infectious Disease Epidemiology and Research (CIDER), School of Public Health and Family Medicine, University of Cape Town, Cape Town, South Africa; 5 Division of Epidemiology and Biostatistics, School of Public Health and Family Medicine, University of Cape Town, Cape Town, South Africa

**Keywords:** HIV and cancer, Epidemiology, South Africa, Antiretroviral era, Cancer risk

## Abstract

**Introduction:**

The impact of South Africa’s high human immunodeficiency virus (HIV) burden on cancer risk is not fully understood, particularly in the context of antiretroviral treatment (ART) availability. We examined national cancer trends and excess cancer risk in people living with HIV (PLHIV) compared to those who are HIV-negative.

**Methods:**

We used probabilistic record linkage to match cancer records provided by the National Cancer Registry to HIV data provided by the National Health Laboratory Service (NHLS). We also used text search of specific HIV terms from the clinical section of pathology reports to determine HIV status of cancer patients. We used logistic and Joinpoint regression models to evaluate the risk and trends in cancers in PLHIV compared to HIV-negative patients from 2004 to 2014. In sensitivity analysis, we used inverse probability weighting (IPW) to correct for possible selection bias.

**Results:**

A total of 329,208 cancer cases from public sector laboratories were reported to the NCR from 2004 to 2014 with the HIV status known for 95,279 (28.9%) cancer cases. About 50% of all the female cancer cases (*n* = 30,486) with a known status were HIV-positive. PLHIV were at higher risk of AIDS-defining cancers (Kaposi sarcoma [adjusted OR:134, 95% CI:111–162], non-Hodgkin lymphoma [adjusted OR:2.73, 95% CI:2.56–2.91] and, cervix [adjusted OR:1.70, 95% CI:1.63–1.77], conjunctival cancer [adjusted OR:21.5, 95% CI:16.3–28.4] and human papilloma virus (HPV) related cancers (including; penis [adjusted OR:2.35, 95% CI:1.85–2.99], and vulva [adjusted OR:1.94, 95% CI:1.67–2.25]) compared to HIV-negative patients. Analysis using the IPW population yielded comparable results.

**Conclusion:**

There is need for improved awareness and screening of conjunctival cancer and HPV-associated cancers at HIV care centres. Further research and discussion is warranted on inclusive HPV vaccination in PLHIV.

## Introduction

In Africa, 25.7 million people currently live with the Human Immunodeficiency Virus (HIV) as of 2017 [1]. In South Africa, approximately 14% of the population was living with HIV in 2017 [2]. Since the introduction of antiretroviral treatment (ART) in 2004, there has been an increase in longevity amongst people living with HIV (PLHIV) in South Africa [3]. With this increase in longevity and the known association between cancer and HIV, the risk for cancer amongst PLHIV has increased. However, the additional risk of cancer that PLHIV in South Africa have compared to those who are HIV negative in the ART era is not fully documented.

Studies in developed countries have shown a higher burden of non-AIDS-defining cancers (NADCs) amongst PLHIV in the ART era particularly, anal, skin, liver and lung cancer [4, 5]. Associated with this is age, race, unavailability of ART in some cases, HIV transmission route, lifestyle related factors and immunosuppression [4, 6–8]. However, not all NADCs have exhibited differential rates before and after ART. For example PLHIV have remained at low risk of colon, breast and prostate cancers, leading to the possibility that not all cancers are associated with immunosuppression [9]. In contrast, developing countries still have a higher burden of AIDS-defining cancers (ADCs), namely Kaposi Sarcoma (KS), cervical cancer (CC), and non-Hodgkin lymphoma (NHL). This is largely due to co-infections with oncogenic viruses and possibly, poor access to HIV care including ART [10–12].

Studies on HIV and cancer done in South Africa have involved HIV cohorts or case control studies which have limited generalization to the general population [13, 14]. The cancer data provided by the National Cancer Registry (NCR) lacks information on HIV status amongst cancer patients as HIV status is not routinely collected in the cancer registry. The South African HIV Cancer Match (SAM) study is a probabilistic record linkage study. It consists of a national HIV cohort created from National Health Laboratory Service (NHLS) HIV laboratory data (CD4 counts, viral load, HIV tests), linked to the NCR data, in order to study cancer risk in HIV positive people [15]. The current study is nested within the SAM study. We aimed to determine the impact of HIV on cancer burden and the cancer risk in PLHIV compared to HIV negative people or the general South African population.

## Methods

### Study setting and design

The NHLS is the largest diagnostic pathology service in South Africa. It provides laboratory and public health services to over 80% of the South African population [16]. This is achieved through a national network of laboratories in all the nine provinces of South Africa. The NHLS’ Corporate Data Warehouse (CDW) is an electronic data repository for all public sector laboratory data. The NCR’s main mandate is pathology-based cancer surveillance with both private and public laboratories legislated to report all cancer cases to the institution. This was a cross sectional study of all cancers diagnosed in public sector laboratories from 2004 to 2014 with HIV data being obtained from the NHLS’ CDW.

### Study population, variables and data sources

We included all records of patients diagnosed with cancer in public healthcare laboratories from 2004 to 2014. Cancer diagnosis was coded according to International Classification of Diseases for Oncology (ICD-O-3) excluding all cancer pre-cursor lesions. Since the source of our HIV data was the NHLS, which services the public sector, we excluded cancer records from the private sector. Our rationale was, if a patient accessed cancer care at a private facility, they were more likely to access HIV care at a private facility as well [17]. From our linkage out of the 335,589 cancer records that were reported from the private sector only 1122 had a known HIV result thus supporting our hypothesis.

An individual was considered HIV positive or negative if the HIV diagnostic test result was positive or negative respectively. If the result was indeterminate or neither positive nor negative, the HIV result was regarded as unknown. In addition, HIV monitoring tests such as HIV viral load and CD4 counts were used to assume an HIV positive status. To supplement the NHLS HIV dataset, repeated text mining was done to extract more HIV results from the clinical section of pathology reports on confirmed cases of cancer reported to the NCR. By definition, text mining refers to the drawing out of important and specific information from a block of text [18]. The text mining process involved the use of key terms used to refer or infer HIV status. The key words used included, “HIV”, “HIV+” “HIV positive” “AIDS”, “haart”, “ART”, “ARV”, “antiretroviral”, anti-retroviral”, “RVD”, “RVD positive”, “retroviral disease”, “immune suppression”, “immunosuppression”, “immuno-suppression”, “acquired immune-deficiency”, “retroreactive”, “immunocompromised”, “HIV reactive”, “CD4”, “regimen 1 treatment”, “reg 1 treatment “Retroviral disease”, “RVD”, “HIV”, “HAART” and “ARV”. From the extracted records a series of samples were taken and reviewed to refine the search terms. Demographic characteristics and potential confounders such as age, gender and race were extracted from the NCR database.

### Data management

The HIV and cancer datasets were linked using the in house CDW probabilistic record linkage algorithm. This algorithm is used to link all the laboratory records that belong to the same individual within the entire NHLS database. The linkage variables include name, surname and date of birth. For records to be considered a match, the first letter of the first names should match and two components of the date of birth must also match. First names and surnames are given the same linkage weights (40% each) and the date of birth contributes 20% of the overall weight. For records with a recorded national identity number, exact matching is done and this is used to validate the probabilistic record linkage. Records that attain a score of 90% and above are considered a match. After linkage, duplicates were removed and private sector cancer records were excluded and a final sample of 329,280 records remained.

Cervical cancer, KS and NHL were classified as ADCs and the rest of the malignancies as NADCs. We also looked at NHL subtypes namely, Burkitt lymphoma, Diffuse large B-cell lymphoma (DLBCL), Diffuse immunoblastic large B-cell lymphoma (DILBCL), follicular lymphoma Not otherwise Specified (NOS) and NHL NOS. The NADCs were grouped into virus-related and virus unrelated cancers. The following were classified as virus-related cancers according to the IARC Monograph Working group assessment; liver cancer (hepatitis viruses), penis, vulva, vagina, anal, oropharynx, larynx and tonsil (Human Papilloma Virus (HPV) other than cervix) and Hodgkin’s lymphoma and nasopharyngeal cancer (Epstein Barr Virus (EBV)) [19]. Although all the ADCs are associated with viruses they were not included in the virus related NADCs category. For descriptive purposes, age was classified as 0–14, 15–19, 20–24, 25–29, 30–34, 35–39, 40–44, 45–49, 50–54, 55–59 and 60 + .

### Data analysis

We determined the characteristics of cancer patients (age, gender, race, cancer type (NADC or ADC) and cancer diagnosis year) by HIV status (positive, negative or unknown) with 95% confidence intervals. To determine the additional risk that PLHIV had of developing specific cancers as per ICD-O-3 coding, logistic regression models were fitted adjusting for age (as a continuous variable), gender (males and females), race (Asian, Black, Coloured and White) and cancer diagnosis year (modelled as a continuous variable).

We assessed trends in cancer risk for selected cancers by plotting yearly crude odds ratios using Joinpoint regression models (Joinpoint Regression Program, Version 4.6.0.0. April, 2018 Statistical Research and Applications Branch, National Cancer Institute). The Joinpoint program allows one to determine if the trend observed is statistically significant or not. In most cases the independent variable is the calendar year. Observed odds ratios (or other parameters such as incidence rate or counts) are joined in straight lines at each time point hence the term joinpoint. The model goes to identify at which time point a significant change in trend is observed as well as the magnitude of the change (Annual Percentage Change (APC)). Permutation tests are then used to select the final model that better describes the change in trends. To determine the contribution of HIV to the cancer burden in South Africa, we calculated Attributable Risk Fractions (ARFs) using adjusted odds ratios as demonstrated by Newson [20].

### Sensitivity analysis

Clinicians are more likely to request an HIV test if the patient is symptomatic, hence creating a selection bias. With high number of missing HIV status, inverse probability weighting (IPW) methods were used as a post-hoc sensitivity analysis to correct for possible selection bias. We created the weights using age, gender, cancer diagnosis year and cancer type similar to the method used by Dryden-Petersen et al. [10].

Analysis was done using Stata version 15 (College Station, TX: StataCorp LP). *P*-values of less than 0.05 were considered to be statistically significant.

## Results

**Fig. 1:**
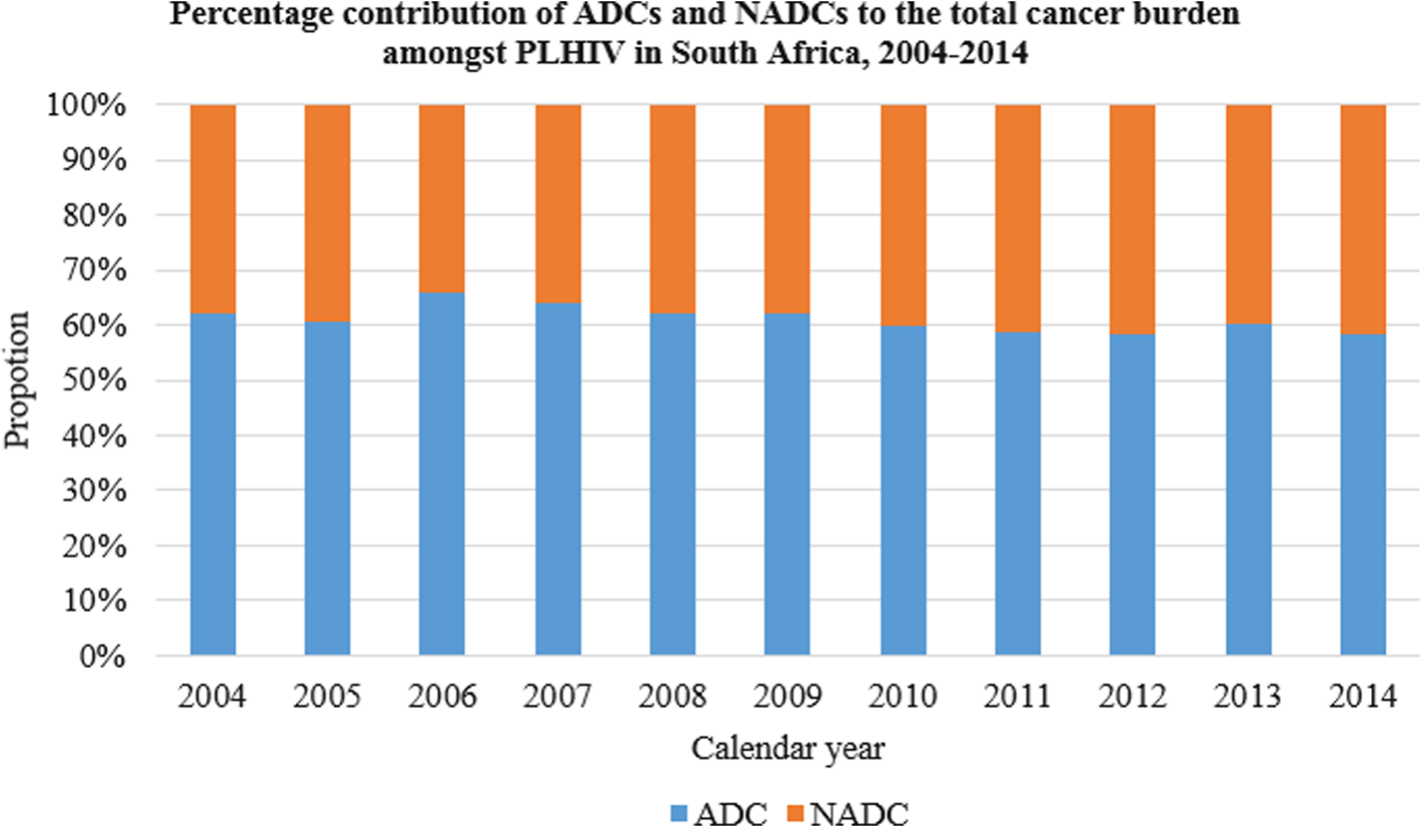
Percentage contribution of ADCs and NADCs to the total cancer burden amongst PLHIV in South Africa, 2004–2014. A comparison of incident cancers by cancer type in PLHIV. Given in the graph is a percentage of the total cancers in PLHIV each year

From 2004 until 2014, a total of 329,208 cancers were reported to the NCR by the public sector laboratories. Probabilistic record linkage identified 90,796 HIV results and through text mining of cancer pathology reports an additional 4483 HIV results were found. Of the 95,279 (28.9%) cancer patients with a known HIV status, 46,951 (14.3%) were HIV positive. Amongst PLHIV, cancer proportions were highest between the ages of 25 and 49 (Table 1 below). In contrast, 37% (*n* = 17,890) of all HIV negative individuals were in the over 60 age group. Across all the HIV status subgroups, the greater proportion of cancers was observed in the Black population at 62.6% (*n* = 206,286). A general increase in cancer proportions was observed for all cancers irrespective of the HIV status by calendar year. Compared to the HIV negative individuals and those with an unknown status, more ADCs were observed in PLHIV. Throughout the study period, ADCs remained constantly higher than NADCs in HIV positive individuals, (Fig. 1 below).

**Table 1:** Characteristics and distribution of public sector cancer cases by HIV status, 2004–2014

	HIV POSITIVE		HIV NEGATIVE		HIV UNKNOWN	
Characteristic	*n* = 46,951	Proportion (95% CI)	*n* = 48,328	Proportion (95% CI)	*n* = 234,001	Proportion (95% CI)
Gender						
Female	30,487	0.65(0.645–0.654)	30,032	0.62(0.617–0.626)	129,051	0.55(0.549–0.554)
Male	16,443	0.35(0.346–0.355)	18,287	0.38(0.374–0.383)	104,413	0.45(0.444–0.448)
Missing	21	0(0–0.001)	9	0(0–0)	538	0(0.002–0.002)
Age						
0–14	590	0.01(0.012–0.014)	2755	0.06(0.055–0.059)	3091	0.01(0.013–0.014)
15–19	338	0.01(0.006–0.008)	582	0.01(0.011–0.013)	1331	0.01(0.005–0.006)
20–24	1317	0.03(0.027–0.03)	742	0.02(0.014–0.016)	2407	0.01(0.01–0.011)
25–29	3915	0.08(0.081–0.086)	1020	0.02(0.02–0.022)	4839	0.02(0.02–0.021)
30–34	6733	0.14(0.14–0.147)	1295	0.03(0.025–0.028)	7822	0.03(0.033–0.034)
35–39	7773	0.17(0.162–0.169)	2250	0.05(0.045–0.048)	10,468	0.04(0.044–0.046)
40–44	7220	0.15(0.151–0.157)	3452	0.07(0.069–0.074)	14,051	0.06(0.059–0.061)
45–49	5812	0.12(0.121–0.127)	5081	0.11(0.102–0.108)	17,996	0.08(0.076–0.078)
50–54	4671	0.1(0.097–0.102)	6291	0.13(0.127–0.133)	22,903	0.1(0.097–0.099)
55–59	3351	0.07(0.069–0.074)	6588	0.14(0.133–0.139)	26,590	0.11(0.112–0.115)
60+	4738	0.1(0.098–0.104)	17,890	0.37(0.366–0.374)	113,501	0.49(0.483–0.487)
Missing	493	0.01(0.01–0.011)	382	0.01(0.007–0.009)	9003	0.04(0.038–0.039)
Race						
Asian	333	0.01(0.006–0.008)	826	0.02(0.016–0.018)	6869	0.03(0.029–0.03)
Black	40,004	0.85(0.849–0.855)	25,335	0.52(0.52–0.529)	140,946	0.6(0.6–0.604)
Coloured	2620	0.06(0.054–0.058)	10,107	0.21(0.206–0.213)	28,793	0.12(0.122–0.124)
White	1548	0.05(0.05–0.054)	1641	0.22(0.212–0.219)	10,115	0.2(0.2–0.204)
Missing	2446	0.03(0.031–0.035)	10,419	0.03(0.032–0.036)	47,279	0.04(0.042–0.044)
Province						
Eastern Cape	3314	0.07(0.068–0.073)	4020	0.08(0.081–0.086)	32,379	0.14(0.137–0.14)
Free State	4364	0.09(0.09–0.096)	5178	0.11(0.104–0.11)	16,773	0.07(0.071–0.073)
Gauteng	20,957	0.45(0.442–0.451)	15,534	0.32(0.317–0.326)	62,004	0.26(0.263–0.267)
Kwazulu-Natal	1434	0.03(0.029–0.032)	712	0.01(0.014–0.016)	35,268	0.15(0.149–0.152)
Limpopo	3661	0.08(0.076–0.08)	553	0.01(0.01–0.012)	15,529	0.07(0.065–0.067)
Mpumalanga	3277	0.07(0.067–0.072)	593	0.01(0.011–0.013)	9390	0.04(0.039–0.041)
North West	2869	0.06(0.059–0.063)	1526	0.03(0.03–0.033)	9700	0.04(0.041–0.042)
Northern Cape	1098	0.02(0.022–0.025)	1396	0.03(0.027–0.03)	6561	0.03(0.027–0.029)
Western Cape	5962	0.13(0.124–0.13)	18,791	0.39(0.384–0.393)	45,466	0.19(0.193–0.196)
Missing	15	0(0–0)	25	0(0–0.001)	932	0(0.004–0.004)
Type of cancer						
NADC	17,604	0.37(0.371–0.379)	34,965	0.72(0.72–0.727)	172,260	0.74(0.734–0.738)
ADC	27,125	0.58(0.573–0.582)	10,288	0.21(0.209–0.217)	42,992	0.18(0.182–0.185)
Primary site unknown	2222	0.05(0.045–0.049)	3075	0.06(0.061–0.066)	18,750	0.08(0.079–0.081)
Cancer diagnosis year						
2004	1026	0.02(0.021–0.023)	1767	0.04(0.035–0.038)	23,656	0.1(0.1–0.102)
2005	2337	0.05(0.048–0.052)	3281	0.07(0.066–0.07)	21,784	0.09(0.092–0.094)
2006	3038	0.06(0.062–0.067)	3678	0.08(0.074–0.078)	23,101	0.1(0.098–0.1)
2007	3616	0.08(0.075–0.079)	3882	0.08(0.078–0.083)	22,376	0.1(0.094–0.097)
2008	4434	0.09(0.092–0.097)	4378	0.09(0.088–0.093)	22,506	0.1(0.095–0.097)
2009	4782	0.1(0.099–0.105)	4839	0.1(0.097–0.103)	21,917	0.09(0.092–0.095)
2010	5194	0.11(0.108–0.113)	4926	0.1(0.099–0.105)	21,279	0.09(0.09–0.092)
2011	5714	0.12(0.119–0.125)	5485	0.11(0.111–0.116)	19,769	0.08(0.083–0.086)
2012	5931	0.13(0.123–0.129)	5749	0.12(0.116–0.122)	21,592	0.09(0.091–0.093)
2013	5719	0.12(0.119–0.125)	5401	0.11(0.109–0.115)	18,881	0.08(0.08–0.082)
2014	5159	0.11(0.107–0.113)	4942	0.1(0.1–0.105)	17,141	0.07(0.072–0.074)

Correcting for age, gender, race, and year of cancer diagnosis, cancer risk was highest in the HIV positive population for all ADCs (Kaposi sarcoma, NHL, and cervical cancer) with an overall adjusted odds ratio of 4.5 (95% CI =4.35–4.65). The NHL subtypes Burkitt’s lymphoma (adjusted OR: 6.48, 95% CI (5.21–8.07)), Diffuse large B-cell lymphoma (DLBCL) (adjusted OR 2.93 95% CI (2.67–3.22)) and Diffuse immunoblastic large B-cell lymphoma (DILBCL) (adjusted OR 12.1 95% CI (9.02–16.3)). Compared to HIV negative individuals, PLHIV were 0.74 times less likely to develop NADCs (adjusted OR: 0.26, 95% CI (0·25–0.26). As a group, virus-related NADCs were not significantly associated with HIV but most of the HPV-associated cancers such as anal, penile, vulva and lip and Hodgkin’s lymphoma (EBV-associated), were high risk in HIV positive individuals [*p* < 0.0001]. Liver cancer, which is associated with hepatitis viruses, was not significantly associated with HIV. People living with HIV were at a higher risk for Squamous Cell Carcinoma (SCC) of the skin, Basal Cell Carcinoma (BCC), eye, and conjunctival cancers (*p* < 0.0001). Non-virus related NADCs were also not associated with HIV. The weighted analysis produced results that were comparable to the complete case analysis (Table 2).

**Table 2:** Odds ratios for cancer in PLHIV by complete case analysis and by weighted analysis

Cancer	Complete case		Weighted	
	Odds Ratio (95% CI)	*p*-value	Odds Ratio (95% CI)	*p*-value
ADC	4.50 (4.35–4.65)	< 0.0001	3.46 (3.33–3.59)	< 0.0001
Kaposi	134 (111–161)	< 0.0001	98.8 (80.9–120)	< 0.0001
Cervix	1.70 (1.63–1.77)	< 0.0001	1.68 (1.60–1.76)	< 0.0001
NHL	2.73 (2.56–2.91)	< 0.0001	2.89 (2.71–3.08)	< 0.0001
Burkitt’s lymphoma	6.48 (5.21–8.07)	< 0.0001	7.83 (6.38–9.62)	< 0.0001
Non-Hodgkins NOS	4.26 (3.4–5.34)	< 0.0001	4.85 (3.88–6.07)	< 0.0001
DLBCL	2.93 (2.67–3.22)	< 0.0001	3.27 (3.00–3.59)	< 0.0001
DILBCL	12.1 (9.02–16.3)	< 0.0001	12.0 (8.91–16.1)	< 0.0001
Follicular	0.65 (0.49–0.87)	0.004	0.73 (0.55–0.98)	0.038
NADC	0.26 (0.25–0.26)	< 0.0001	0.36 (0.35–0.38)	< 0.0001
Virus-related NADC	0.75 (0.71–0.78)	< 0.0001	0.77 (0.73–0.82)	< 0.0001
Anus	1.63 (1.33–2.00)	< 0.0001	1.61 (1.30–1.99)	< 0.0001
Hodgkin’s lymphoma	1.22 (1.09–1.37)	< 0.0001	1.43 (1.27–1.61)	< 0.0001
Liver	0.45 (0.39–0.53)	< 0.0001	0.51 (0.42–0.61)	< 0.0001
Vulva	1.94 (1.67–2.25)	< 0.0001	1.82 (1.58–2.10)	< 0.0001
Vagina	0.83 (0.67–1.03)	0.09	0.88 (0.70–1.11)	0.29
Penis	2.35 (1.85–2.99)	< 0.0001	2.06 (1.63–2.61)	< 0.0001
Lip, oral cavity and Pharynx (C00-C14)	0.55 (0.50–0.59)	< 0.0001	0.67 (0.61–0.73)	< 0.0001
Gum	0.74 (0.41–1.36)	0.34	0.90 (0.46–1.76)	0.76
Lip	2.72 (1.71–4.32)	< 0.0001	3.47 (1.95–6.16)	< 0.0001
Mouth	0.56 (0.49–0.66)	< 0.0001	0.63 (0.53–0.75)	< 0.0001
Naso-oropharynx	0.48 (0.42–0.56)	< 0.0001	0.57 (0.48–0.68)	< 0.0001
Salivary gland	0.65 (0.51–0.82)	< 0.0001	0.71 (0.54–0.93)	0.01
Tongue	0.51 (0.43–0.60)	< 0.0001	0.57 (0.47–0.70)	< 0.0001
Virus-unrelated NADC	0.30 (0.29–0.31)	< 0.0001	0.43 (0.42–0.45)	< 0.0001
BCC	1.27 (1.08–1.49)	< 0.0001	1.39 (1.17–1.67)	< 0.0001
Bladder	0.74 (0.61–0.89)	< 0.0001	0.86 (0.69–1.08)	0.2
Bone	0.32 (0.26–0.40)	< 0.0001	0.41 (0.30–0.54)	< 0.0001
Brain	0.27 (0.22–0.34)	< 0.0001	0.34 (0.26–0.43)	< 0.0001
Colorectal	0.43 (0.38–0.47)	< 0.0001	0.55 (0.48–0.62)	< 0.0001
Eye	5.9 (5.11–6.82)	< 0.0001	7.73 (6.60–9.05)	< 0.0001
Conjunctiva	21.5 (16.3–28.4)	< 0.0001	20.8 (15.2–28.5)	< 0.0001
Haematology	0.49 (0.37–0.65)	< 0.0001	0.58 (0.43–0.80)	< 0.0001
Kidney	0.18 (0.15–0.21)	< 0.0001	0.23 (0.19–0.29)	< 0.0001
Larynx	0.56 (0.49–0.64)	< 0.0001	0.62 (0.53–0.72)	< 0.0001
Leukaemia	0.25 (0.23–0.28)	< 0.0001	0.33 (0.30–0.37)	< 0.0001
Lung	0.52 (0.48–0.57)	< 0.0001	0.62 (0.56–0.68)	< 0.0001
Melanoma	0.77 (0.63–0.93)	0.01	0.95 (0.76–1.20)	0.69
Mesothelioma	0.50 (0.34–0.75)	< 0.0001	0.51 (0.32–0.80)	< 0.0001
Myeloma	0.61 (0.52–0.71)	< 0.0001	0.75 (0.63–0.89)	< 0.0001
Oesophagus	0.57 (0.52–0.64)	< 0.0001	0.58 (0.52–0.66)	< 0.0001
Pancreas	0.43 (0.33–0.58)	< 0.0001	0.60 (0.42–0.84)	< 0.0001
SCC Skin	1.83 (1.64–2.04)	< 0.0001	1.88 (1.67–2.11)	< 0.0001
Skin	0.95 (0.77–1.16)	0.6	1.11 (0.87–1.41)	0.41
Small intestines	0.33 (0.23–0.48)	< 0.0001	0.35 (0.24–0.53)	< 0.0001
Stomach	0.43 (0.37–0.49)	< 0.0001	0.58 (0.49–0.70)	< 0.0001
Thyroid	0.75 (0.59–0.95)	0.02	1.01 (0.74–1.37)	0.97
Uterus	0.38 (0.34–0.43)	< 0.0001	0.44 (0.39–0.50)	< 0.0001
Breast	0.43 (0.41–0.45)	< 0.0001	0.50 (0.47–0.53)	< 0.0001
Placenta	0.85 (0.58–1.24)	0.39	1.05 (0.70–1.56)	0.83
Prostate	0.85 (0.76–0.95)	< 0.0001	0.95 (0.84–1.08)	0.46
Testis	0.37 (0.26–0.53)	< 0.0001	0.48 (0.32–0.74)	< 0.0001
Poorly specified histology at any site	0.82 (0.77–0.88)	< 0.0001	0.95 (0.88–1.03)	0.2

*OR* Odds ratios determined using logistic regression models adjusting for age, gender, race, province and year of cancer diagnosis, *NHL* non-Hodgkin’s lymphoma, *DLBCL* Diffuse large B-cell lymphoma, *DILBCL* Diffuse Immunoblastic Large B-cell lymphoma, *NOS* Not Otherwise Specified, *BCC* Basal cell carcinoma, *ADC* AIDS defining cancer, *NADC* non-AIDS defining cancer, *SCC skin* squamous cell carcinoma of the skin, *Virus-related NADCs* liver cancer (hepatitis viruses), penis, vulva, vagina, anal, lip, mouth, gum, salivary gland and tonsil (Human Papilloma Virus (HPV) associated malignancies other than cervix), and Hodgkin’s lymphoma and nasopharyngeal cancer (Epstein Barr Virus (EBV)). The weighted analysis included inverse probability weights estimated from known HIV status age, gender, cancer diagnosis and cancer diagnosis year

**Fig. 2:**
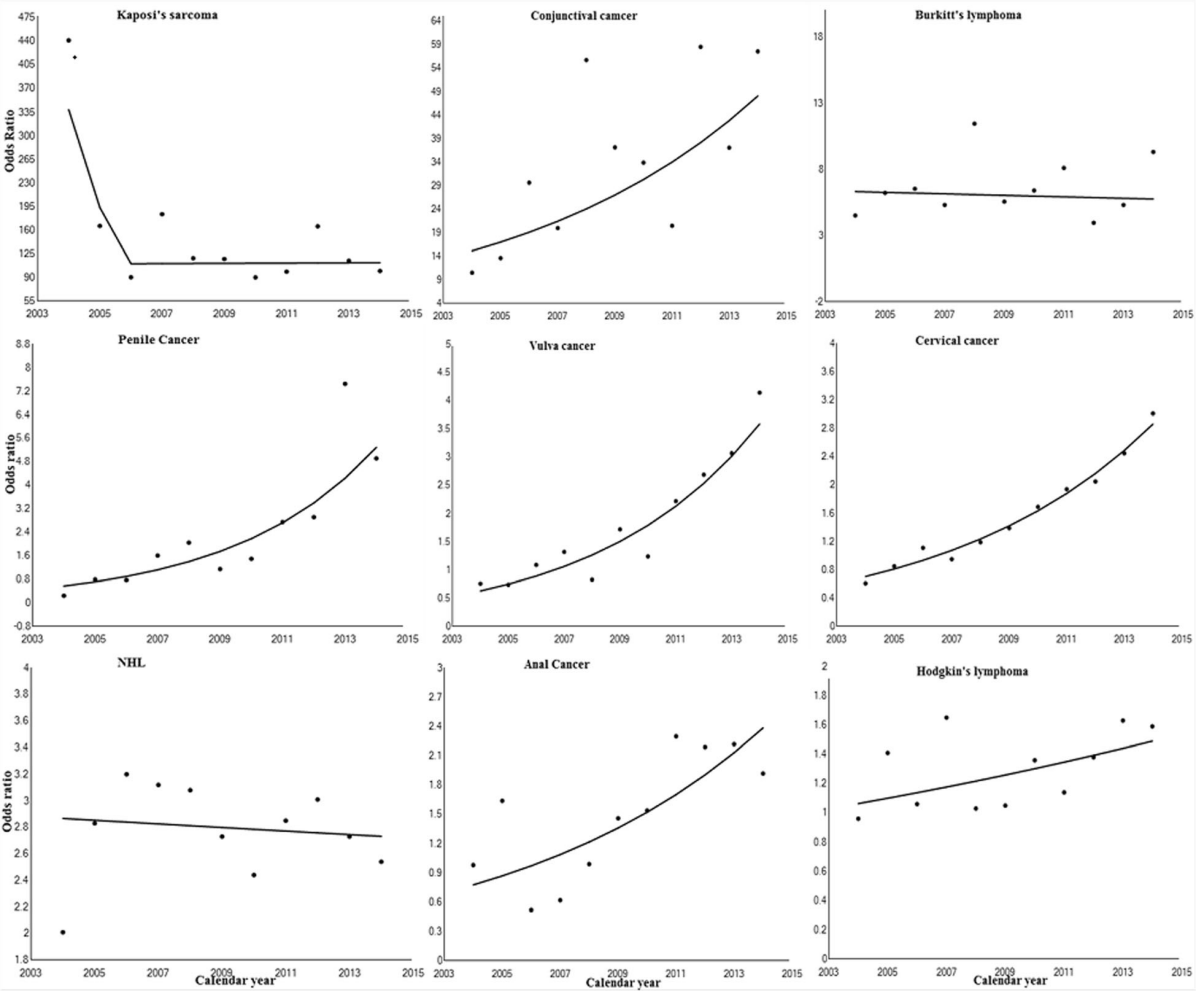
Trends in cancer risk for selected cancers amongst PLHIV in the South African public health sector, 2004–2014. The line graphs were fitted in Joinpoint using crude odds ratios (dots). The annual percentage change in odds ratios was significant (*p*-value < 0.05) for all cancers selected for in-depth analysis of trends except for Kaposi sarcoma, Burkitt’s lymphoma, NHL and Hodgkin’s lymphoma

Trends in cancer risk for selected individual cancers varied, with significant increases observed for cervix, anus, vulva, conjunctiva and penis from 2004 to 2014 in PLHIV (Fig. 2 below). Although the APC was not significant for Kaposi sarcoma, there was a substantial decrease in risk between 2004 and 2006 with no changes observed thereafter. Prior to 2011, there was no significant difference in risk for anal, vulva and penile cancers between those who were HIV negative and PLHIV but significant increases were observed after 2011. For Burkitt’s lymphoma and NHL, whilst the risk was higher in PLHIV, there was relatively no change over the study period. Although insignificant, the trend line for Hodgkin’s lymphoma was suggestive of an increase in cancer risk.

**Fig. 3:**
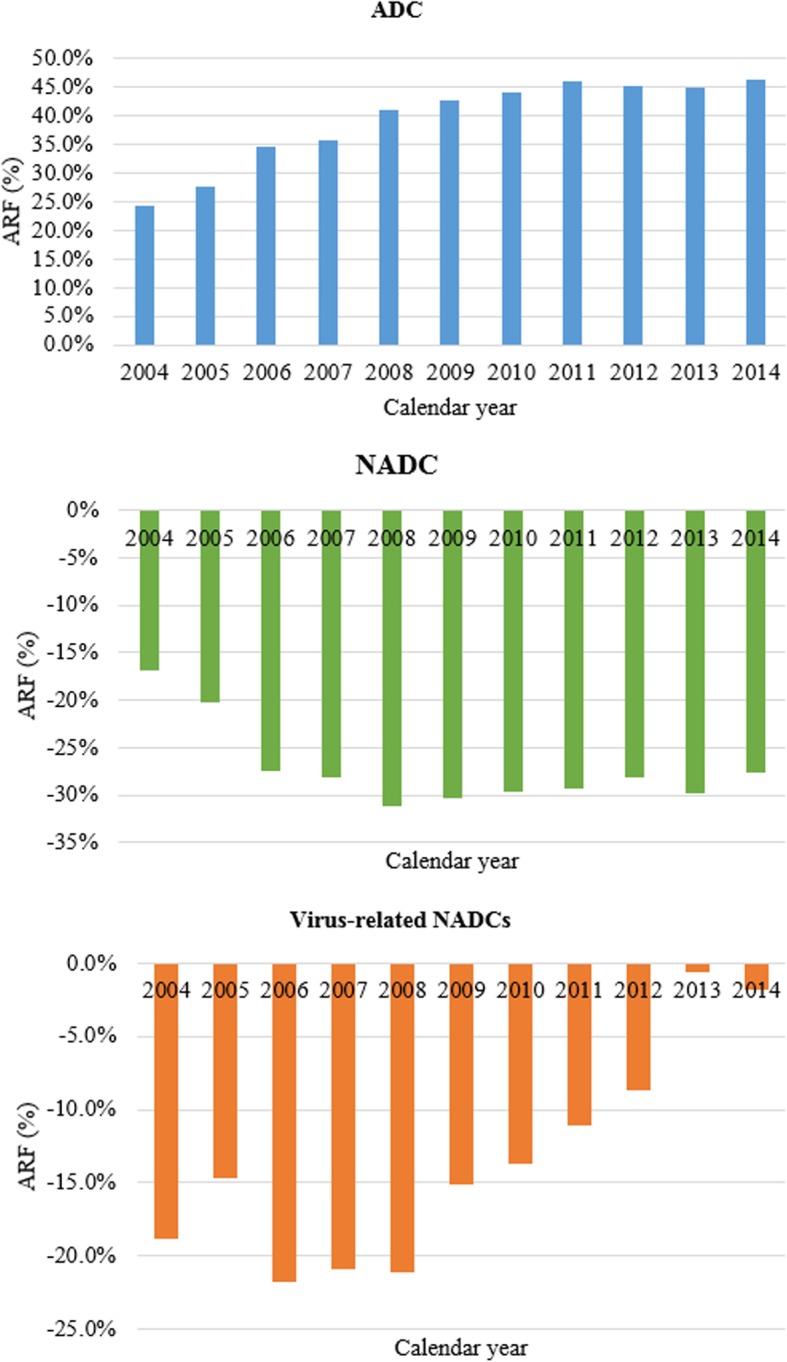
Trend in Attributable risk fractions amongst PLHIV in the South African public health sector, 2004-2014. Using adjusted odds ratios adjusting for age, gender, race, year of cancer diagnosis, and Province. ARF = Attributable Risk Fraction. ADC = AIDS defining cancer (includes Kaposi sarcoma, non-Hodgkin’s lymphoma and cervical cancer). NADCs = Non-AIDS Defining Cancers. Virus-related NADCs = liver cancer (hepatitis viruses), penis, vulva, vagina, anal, lip, mouth, gum, salivary gland and tonsil (Human Papilloma Virus (HPV) associated malignancies other than cervix), and Hodgkin’s lymphoma and nasopharyngeal cancer (Epstein Barr Virus (EBV)

There was no shift in burden between ADCs and NADCs observed amongst incident cancers in PLHIV (Fig. 1). Using weighted estimates of the odds ratio, 41% of all ADCs reported between 2004 and 2014 were attributable to HIV. The contribution of HIV on ADCs increased by 22% within the study period (Fig. 3). No particular contribution by HIV towards NADCs as a whole was noted, given the negative ARFs. The same was true for the category virus-related NADCs (Fig. 3), HIV did not seem to contribute to the burden of virus related NADCs amongst PLHIV in the public sector. However, the “protective” effect of HIV has been waning overtime.

## Discussion

Over the period 2004–2014 (ART era), the risk of all ADCs and some virus-related NADCs was higher amongst HIV positive individuals compared to those who were HIV negative. The strongest association was observed between KS [adjusted OR: 134, 95% CI 111–161], conjunctival cancer [adjusted OR: 21.5, 95% CI 16.3–28.4] as well as Burkitt’s lymphoma [adjusted OR: 6.48, 95% CI 5.21–8.07]. Amongst the virus-related NADCs, HPV-associated cancers such as lip, anal, penile and vulva cancer had the strongest associations with HIV. Compared to those who were HIV negative, squamous cell carcinoma of the skin (SCC), basal cell carcinoma (BCC) and conjunctival cancer were the only virus-unrelated NADCs that were significantly associated with HIV. Over time, amongst PLHIV a significant upward trend in risk was observed for cancer of the conjunctiva and anogenital cancers, including cervix.

The spectrum of cancers observed in this study was comparable to what has been observed in other African countries. Similar to a case control study by Stein et al. conducted before ART was available, the risk of KS, cervical cancer, NHL, anogenital cancers other than cervix and SCC skin was elevated amongst PLHIV in our present study [21]. For KS, the risk was higher compared to the one reported by Stein et.al (adjusted OR: 50.4, 95% CI 34.2–74.3) [21]. In both our study and the Stein pre-ART study, the odds ratios were adjusted for age, gender, race and year of diagnosis, which allowed for comparability. Possible explanations for the higher risk in our study is that, until 2016 when the universal test and treat policy was adopted in South Africa, treatment initiation was dependent on CD4 count [22]. In 2004, ART became freely available in the public sector with patients who had CD4 counts of less than 200 cell/μl or in the WHO stage IV of disease being eligible for treatment [23]. Patients were also evaluated to determine if they were psychological fit to receive the treatment. In 2010 in addition to the 2004 recommendations, those who had a co-infection with TB were also automatically eligible for the free ART [24]. In 2011, the criteria were then expanded to include all patients who had a CD4 count of less than 350 cells/μl [24]. These CD4 count thresholds led to a high proportion of immunosuppressed individuals with a high burden of disease, a risk factor for ADCs [14, 22]. As a result, the risk of KS remained elevated even after ART introduction. Moreover, it is possible that the pick-up rate of KS at HIV clinics improved with the expansion of ART and improvements in HIV treatment policies in South Africa hence the greater strength of association observed. Despite the high risk reported for KS in our study, it was lower than reported in other studies particularly those done in the developed countries [4, 25]. In South Africa, the prevalence of Human Herpes Virus (HHV8) was high even before the HIV era, therefore creating a high KS background risk [26]. In addition to this, clinical diagnosis of KS is quite prevalent in the African context with no biopsies or other samples being sent to the laboratory [27]. Therefore, under-reporting of KS to the pathology-based cancer registry may have been possible.

In contrast, the risk reported in our study for NHL (adjusted OR: 2.73, 95% CI 2.56–2.91) was lower than the one reported by Stein et.al. (adjusted OR: 6.1, 95% CI 4.4–8.4) which points to a possible reduction in risk of NHL after the introduction of ART [21]. There was no change noted before and after ART in overall cervical cancer risk although an upward trend was observed in the ART era. This is in line with other reports from Africa with various reasons being put forth to account for the increase in cervical cancer risk even with the introduction of ART. These include advanced disease upon ART initiation and older age [28, 29]. Another theory that has been put forward is the lack of a relationship between cervical cancer risk and immunosuppression. Some studies have demonstrated that low CD4 counts do not necessarily amount to increased risk of cervical cancer and other HPV-related cancers [29]. As such, restoration of immunity with ART will not necessarily lead to a reduced risk of cervical cancer. In addition, the prevalence of HPV (a known risk factor for cervical cancer) is higher amongst women living with HIV [29, 30]. Possible co-infection with HPV has also been highlighted in this study with increased risk amongst PLHIV observed for HPV associated cancers such as vulva, anus, penis and lip.

Besides the ADCs and HPV related cancers, we observed other additional cancers were strongly associated with HIV in the ART era. Compared to HIV negative individuals, the risk of conjunctival cancer, Hodgkin’s lymphoma and BCC was also higher in PLHIV in our study. Before ART, there were no reports of conjunctival cancer and BCC as being high risk amongst PLHIV in South Africa [21]. The association between conjunctival cancer and HIV has been reported in Africa [29, 31]. High rates of solar radiation and unproved associations with HPV have been cited as possible reasons why this cancer is common in Sub-Saharan Africa compared to other parts of the world [29]. Like SCC skin, we observed stronger associations between HIV and BCC. Reports have linked age and white race to higher BCC risk in PLHIV with immunosuppression and increased viral loads only being linked to SCC skin [32, 33]. On the other hand PLHIV were less likely to develop virus-unrelated cancers such as breast and prostate which is in line with the literature [4, 7, 25]. Lower risks were also observed for lung and liver cancers in PLHIV consistent with the results reported by Stein et al. but contrary to other reports especially those done in resource rich areas [4, 7, 9]. In the resource rich countries, there is a higher prevalence of lifestyle related factors such as smoking which results in lung cancer and increased alcohol intake which results in liver cancer in HIV cohorts [7]. In our study, it is still uncertain why the liver and lung cancer risk was lower in PLHIV compared to HIV negative individuals.

In the ART era, different cancer trends have been observed, with ADCs decreasing upon ART introduction in other settings [6, 9, 25]. In particular, KS has declined with the introduction and expansion of ART hence supporting the association between this cancer and immunosuppression [6, 25, 34]. In our study following the initial drop in KS risk after ART introduction in 2004, there has not been a significant change in risk amongst PLHIV in the ART era [10]. This is similar to what was reported in a recent study done in Botswana which demonstrated a decrease in KS risk with ART introduction but no significant change with increased roll out of ART [10]. The arguments for this are similar to the reasons why KS risk was reported as higher in our study compared to the pre-ART era, which include HIV treatment policies and improved pick-up rate. The trend in NHL risk exhibited a slight but insignificant decrease over the 11-year period. Whilst some studies have shown decreasing trend in NHL in the ART era in PLHIV others have shown stable trends even with the increased rollout of ART [10, 25]. This has largely been because of Burkitt’s lymphoma as its incidence has remained constant even in the ART era.

Also showing increasing trends in the ART era were most HPV related anogenital cancers (cervix, anus, penis and vulva). Although anal cancer is on the rise, the risk reported is not as high as observed in developed countries. This is possibly due to the difference in HIV epidemiology between South Africa and developed countries. In the latter, the main mode of HIV transmission is men who have sex with men (MSM) through receptive anal sex where as in South Africa, HIV transmission is mainly heterosexual [4, 5]. Co-infection with HPV is higher amongst people living with HIV with the routes of transmission being similar to HIV [35]. Both anal and cervical cancer are associated with HPV, but the different transmission routes will result in more cervical cancer in the African context and more anal cancer in developed countries.

This was the first nationwide study to compare cancer risk amongst the HIV positive and HIV negative people in the ART era. Laboratory confirmation of both cancer and HIV allowed for high specificity of HIV and cancer diagnosis. Although a greater proportion of the HIV status was unknown, the methods used to ascertain HIV status such as probabilistic record linkage and text search ensured that we extracted and matched most of the available HIV records. In addition, probabilistic record linkage allowed us to identify records belonging to the same individual even in the absence of a unique identifier. The greater percentage of black population with HIV and cancer was reflective of the HIV epidemic in South Africa as well as patterns of access to public health services. In addition to this, the use of IPW allowed for assessment of the risk estimates given the possible selection bias due to the high proportion of missing HIV status. The conclusions from the weighted analysis (IPW) were comparable with the complete case analysis. Moreover, women were well represented with enough numbers for cancers that are common in females to be fully analysed.

Despite all these strengths, our study had limitations. Due to its laboratory-based surveillance system, the NCR underreports some cancers that are diagnosed clinically or radiologically like lung and liver cancers. This might potentially result in misrepresentation of association between HIV and these cancers. Although probabilistic record linkage allowed for matching, in the absence of a unique identifier there is still room for some false matches. The national unique identifier remains the gold standard. Another limitation of our study was overrepresentation of the HIV positive individuals. Doctors are more likely to note down the HIV status of a patient if the patient is tested positive. In addition to this, specific cancers such as KS and other symptoms that are known to be associated with HIV are more likely to prompt a clinician to request an HIV test to be done on the patient [36]. This will result in a higher HIV testing and subsequently higher HIV prevalence compared to the general population. Therefore, with the text mining of doctors’ clinical notes in pathology-reports, we were more likely to pick up those that were tested positive than those that were tested negative or never tested. As such, our study also shares the same limitations as proportionate incidence ratio studies. The increased risk observed may be a reflection of a higher HIV prevalence resulting in more cancer cases that are associated with HIV in our study population compared to that in the general population. The evaluation of cancer risk in PLHIV as a function of time was not possible in this study. However, through the SAM study determination of cancer risk with a person-time denominator will be possible. Data on other potential confounders such as lifestyle patterns (smoking, alcohol intake, diet and exercise) and other opportunistic infections was also not available. Access to this information would have possibly made the results more robust.

## Conclusion

PLHIV have a higher risk for all ADCs and most virus-related NADCs. The risk of anogenital cancers and conjunctival cancer continues to rise in the ART era and suggests that, ART alone is inadequate in reducing cancer in PLHIV. Most of these cancers are HPV-related. Targeted public health interventions for HPV such as screening and expansion of HPV vaccination (for cervical cancer) amongst PLHIV are essential in reducing the burden. To consolidate these efforts, ART expansion and availability as well as retention in care should be strengthened. With the introduction of universal ART treatment in 2016, further decreases in ADCs are expected provided individuals report to health care centres before the HIV disease has advanced.

## Abbreviations

ADC: AIDS defining cancer
ART: Antiretroviral treatment
BCC: Basal cell carcinoma
HIV: Human immunodeficiency virus
HPV: Human Papilloma Virus
IPW: Inverse probability weighting
KS: Kaposi sarcoma
NADC: Non-AIDS defining cancer
NHLS: National Health Laboratory Service
PLHIV: People living with HIV
SAM: South African HIV Cancer Match
SCC: Squamous cell carcinoma
